# Multi-electrode array study of neuronal cultures expressing nicotinic β2-V287L subunits, linked to autosomal dominant nocturnal frontal lobe epilepsy. An *in vitro* model of spontaneous epilepsy

**DOI:** 10.3389/fncir.2014.00087

**Published:** 2014-07-24

**Authors:** Francesca Gullo, Irene Manfredi, Marzia Lecchi, Giorgio Casari, Enzo Wanke, Andrea Becchetti

**Affiliations:** ^1^Department of Biotechnology and Biosciences, University of Milano-BicoccaMilano, Italy; ^2^Center for Translational Genomics and Bioinformatics, Vita-Salute San Raffaele University and San Raffaele Scientific InstituteMilano, Italy

**Keywords:** β2-V287L, carbamazepine, GABA_A_, MEA, nAChR

## Abstract

Autosomal dominant nocturnal frontal lobe epilepsy (ADNFLE) is a partial sleep-related epilepsy which can be caused by mutant neuronal nicotinic acetylcholine receptors (nAChR). We applied multi-electrode array (MEA) recording methods to study the spontaneous firing activity of neocortical cultures obtained from mice expressing or not (WT) an ADNFLE-linked nAChR subunit (β2-V287L). More than 100,000 up-states were recorded during experiments sampling from several thousand neurons. Data were analyzed by using a fast sliding-window procedure which computes histograms of the up-state durations. Differently from the WT, cultures expressing β2-V287L displayed long (10–32 s) synaptic-induced up-state firing events. The occurrence of such long up-states was prevented by both negative (gabazine, penicillin G) and positive (benzodiazepines) modulators of GABA_A_ receptors. Carbamazepine (CBZ), a drug of choice in ADNFLE patients, also inhibited the long up-states at micromolar concentrations. In cultures expressing β2-V287L, no significant effect was observed on the action potential waveform either in the absence or in the presence of pharmacological treatment. Our results show that some aspects of the spontaneous hyperexcitability displayed by a murine model of a human channelopathy can be reproduced in neuronal cultures. In particular, our cultures represent an *in vitro* chronic model of spontaneous epileptiform activity, i.e., not requiring pre-treatment with convulsants. This opens the way to the study *in vitro* of the role of β2-V287L on synaptic formation. Moreover, our neocortical cultures on MEA platforms allow to determine the effects of prolonged pharmacological treatment on spontaneous network hyperexcitability (which is impossible in the short-living brain slices). Methods such as the one we illustrate in the present paper should also considerably facilitate the preliminary screening of antiepileptic drugs (AEDs), thereby reducing the number of *in vivo* experiments.

## Introduction

Autosomal dominant nocturnal frontal lobe epilepsy (ADNFLE) is a mendelian partial epilepsy which belongs to the subgroup of familial focal epilepsies with autosomal dominant transmission pattern, which includes the familial temporal lobe epilepsy and familial focal epilepsy with variable foci (Engel, 2001). Hundreds of ADNFLE families have been identified, but the exact incidence of the disease is currently unknown because the genetic analysis of many candidate families is incomplete and because of the possibility of misdiagnosis (Picard and Brodtkorb, 2008). ADNFLE is characterized by clusters of hyperkinetic seizures, mostly occurring during stage II of sleep. Attacks originate from the frontal lobe, and usually begin in childhood (Scheffer et al., 1995; Picard et al., 2000). Sudden arousals are also typical of ADNFLE and cognitive and psychological alterations may accompany the epileptic phenotype (Picard et al., 2009). ADNFLE is a good model of the more common sporadic cases of nonlesional NFLE, because the clinical and electroencephalographic features are similar (Picard and Brodtkorb, 2008). Pharmacological treatment, especially with Carbamazepine (CBZ), often control the major symptoms. However, as in many other epilepsies, approximately 30% of the patients remain unresponsive to therapy (Picard and Brodtkorb, 2008). About 10–12% of the ADNFLE families bear mutations on genes coding for nicotinic acetylcholine receptors (nAChR) subunits (Steinlein et al., 1995; De Fusco et al., 2000; Phillips et al., 2001; Aridon et al., 2006). Another gene recently implicated in this pathology is KCNT1, coding for a sodium-gated K^+^ channel (Heron et al., 2012). The alterations produced by the ADNFLE-linked mutations *in vivo* are subtle, but are generally attributed to neocortical hyperexcitability in the frontal regions (Mann and Mody, 2008; Becchetti, 2012; Wallace and Bertrand, 2013).

Several knock-in models have been produced to study ADNFLE in mice (Klaassen et al., 2006; Teper et al., 2007; Xu et al., 2010) and rats (Zhu et al., 2008). The phenotypes observed reflect some of the features of the human disease, although the alterations appear to be strain-dependent. None of these strains shows major morphological changes in the brain. The neurophysiological evidence, although incomplete, points to alterations in release of neurotransmitters, especially GABA (Klaassen et al., 2006; Teper et al., 2007; Zhu et al., 2008). Recently, Manfredi et al. (2009) developed murine strains which conditionally express the ADNFLE-linked β2-V287L subunit under control of the tetracycline promoter (TET-off system). Expression of β2-V287L can be silenced by doxycycline administration. Untreated mice display a spontaneous epileptic phenotype, with seizures generally occurring during periods of increased delta wave activity, during the light period (corresponding to the resting-sleeping phase in mice). Silencing β2-V287L in adult mice cannot revert the epileptic phenotype. Conversely, mice in which the transgene is silenced between E1 to P15 show no evidence of the epileptic phenotype, even after the mutant subunit is re-expressed. Hence, the mutant subunit needs to be expressed during sensitive phases of brain development for seizures to develop, suggesting that critical stages of synaptic stabilization are implicated in the pathogenesis of ADNFLE.

In general, chronic animal models of epilepsy can be produced by application of chemical convulsants (Avanzini et al., 2008), or by producing transgenic strains, such as those mentioned above, which reproduce some of the features of genetic epilepsies (Cuppola and Moshé, 2012). From both of these, *in vitro* preparations such as brain slices or primary neuronal cultures are used for detailed mechanistic and pharmacological studies. However, spontaneous epileptiform activity is rarely reproduced *in vitro*, particularly with neocortical cells. The scarcity of good *in vitro* models of spontaneous epilepsy hampers the study of pathological excitability. Analogous difficulties apply to the study of antiepileptic drugs (AEDs). Most of them are ion channel inhibitors which can be rather promiscuous in their targets (Macdonald and Rogawski, 2008; Di Resta and Becchetti, 2013). Again, the complex mechanisms of action of these compounds are best studied *in vitro*. However, brain slice preparations are generally too short-living to allow (i) investigating the medium- and long-term changes determined by AEDs; and (ii) carrying out extensive drug screening.

We here establish an *in vitro* model of ADNFLE showing spontaneous hyperexcitability. To this purpose, we used Manfredi’s strains not treated with doxycycline, to compare the action potential firing activity of neocortical cultures from mice expressing or not β2-V287L. Cell firing was recorded by using multi-electrode array (MEA) platforms, which permit long-term continuous extracellular recording of the main excitability features (Gullo et al., 2009, 2012). Long-term neuronal cultures are particularly suitable to study the global tonic effects of physiological ligands and AEDs on network excitability (Gullo et al., 2010; Puia et al., 2012). If necessary, they permit to reconstruct *in vitro* the connectivity pathways between different brain regions, by co-cultured slices (Dossi et al., 2013). The neuronal networks from ADNFLE mice produced spontaneous epileptiform activity, characterized by prolonged up-states interspersed among the normal up- and down-states. Besides the general relevance of an *in vitro* model showing spontaneous hyperexcitability, our cultures permit to distinguish the effects of β2-V287L expression on synaptic formation and local synaptic activity, from the effects observed *in vivo*, i.e., in the context of extracortical input, and particularly innervation of the neocortex by thalamocortical and brain stem nuclei.

## Materials and methods

### Murine strains

We used the S3 and S5 lines of double transgenic mice [FVB-Tg(tTA:*Chrnb2*^V287L^], which express β2-V287L and the tetracycline-controlled transcriptional activator tTA (Manfredi et al., 2009). When the transgene is not silenced by doxycyclin, both lines show spontaneous seizures, more prolonged in S3 (25 s on average). Seizures mostly (75%) occurred during the light period, which in mice corresponds to the resting sleeping phase. The vast majority (>90%) of these seizures took place during periods of increased delta (0.5–4 Hz) electroencephalographic activity (for details, see Manfredi et al., 2009). Animals were housed in SPF conditions on a 12-h light-dark cycle, at 21 ± 1°C, 55 ± 10% humidity and free access to food and water. Mice genotyping was carried out as previously described (Manfredi et al., 2009). Experiments were carried out by following the Principles of Laboratory Animal Care (directive 86/609/EEC). All efforts were made to minimize the number of animals used.

### Cell cultures

Primary cultures of neocortical neurons were prepared from individual post-natal (P1–P3) mice, as previously described (Gullo et al., 2009). As controls, we used the littermates not expressing the transgene [FVB-Tg(PrnP-tTA)], therefore the procedure sometimes used with MEA recording of pooling the cultures from different individuals to decrease intrinsic variability (Wagenaar et al., 2006) could not be applied here. In brief, after dissociation cells were plated at densities of 600–900 × 10^3^ cells/ml on MEA dishes (3–4 per animal) coated with polyethyleneimine 0.1% (wt/vol) and laminin 20 μg/ml. Cultures were maintained in neurobasal medium, containing B27 (InVitrogen, Italy), glutamine (1 mM) and basic fibroblast growth factor (10 ng/ml), in 5% CO_2_ at 37°C. The average final cell density is in the order of 3000 per mm^2^.

### Chemicals and drugs

Unless otherwise indicated, chemicals and drugs were purchased from Sigma-Aldrich (Italy). Stock solutions for gabazine, penicillin-G diazepam, and midazolam were prepared in distilled water and kept at −20°C. CBZ was diluted in dimethyl sulfoxide (100 mM). Before each experiment, stock solutions were diluted as appropriate with the MEA culture medium and the final added volume was always less than 1% (1‰ for CBZ) of the total volume bathing the neurons. Washout was carried out with a solution pre-conditioned by the same network under control conditions.

### MEA recording, waveform acquisition and sorting

We generally record by using MEA dishes (60-electrodes; Multichannels System, Germany) with no fewer than 25 active electrodes and 60 units. The network activity was recorded continuosly at 36°C in CO_2_-controlled incubators for up to 12 h. The registration can thus be considered at the steady state (Gullo et al., 2010). We generally check the activity of our cultures between 9 and 22 days *in vitro* (DIV), as a progressive change of network activity is known to occur after plating. In agreement with Wagenaar et al. (2006) we routinely observe that such process is quicker up to 10–12 DIV (see also Gullo et al., 2009). Therefore, the results here presented refer to cultures aged 13–17 DIV, so that the variability caused by culture “maturation” is maintained within reasonable limits. Analog signals were sampled at 40 kHz with MEA-1060BC or 1060INV pre-amplifiers (bandwidth 1–8000 Hz, Multichannel Systems, Germany) connected to a MEA Workstation (bandwidth 100–8000 Hz, Plexon Inc., USA). Data were sorted into timestamp files by the MEA Workstation Sorter software (MEAWS) and cleaned of artifacts using the OFFLine Sorter program (Plexon Inc., USA). The average MEA spike waveform firing rate in the controls was 68 ± 9.2 Hz (*n* = 18), consistent with literature (Wagenaar et al., 2006). The MEAWS capture acquisition procedure was carried out in a window of 1.2 ms, according to a mixed amplitude/duration criterion (Gullo et al., 2009). The electrodes responding irregularly during the experiments were excluded from the analysis. Subsequently, to avoid artifacts, threshold was readjusted and signals were cleaned of spikes whose inter-spike interval was shorter than the pre-fixed 2.5 ms refractory period, by the OFFLine Sorter program (Plexon Inc.). To sort the waveforms with a principal component analysis (PCA) and for multi-unit electrodes, we applied a spike removal procedure with a Mahalanobis threshold in the range 1.8–1.4. Concomitantly, we checked that the *p*-value of multivariate ANOVA sorting statistics among the identified units was <0.01. When this procedure led to excessive spike invalidation, the spikes invading the adjacent unit ellipsoids were manually removed (Gullo et al., 2009, 2010). Overall, the percentage of events excluded was always less than 10%.

### Neuronal cluster identification

For each identified unit and each burst, we computed in defined time segments the autocorrelation function (ACF), the burst duration (BD), the spike number (SN), the spike rate (SR), the intra-burst spike rate (IBSR), the inter-burst intervals (IBIs) and the Fano factor (FF; Teich, 1989; Baddeley et al., 1997). Units were classified with an unsupervised learning approach consisting of FF-based data reducing PCA (Becchetti et al., 2012), followed by the *K*-means clustering procedure (Duda et al., 2000). We did not cluster our units based on spike-width (Constantinidis and Goldman-Rakic, 2002), as this was not distributed bimodally (Becchetti et al., 2012). Conversely, the FF-based clustering gave a distinct bimodal pattern in the FF, BD, SN and IBSR histograms, which clearly identifies the two main clusters of neurons. Cluster definition was refined by using an outlier removal procedure which discards the units whose Mahalanobis distance from the centroid of the cluster is greater than a fixed threshold (we used 1.4). As previously described (Becchetti et al., 2012), this procedure normally identifies two clusters composed, respectively, of variable numbers of excitatory (~50–80) and inhibitory (~15–25) neurons, whose ratio broadly fits the one observed in the neocortex (Sahara et al., 2012).

### Burst analysis

Bursts were analyzed as previously described (Gullo et al., 2009, 2010). Bursts composed of more than two spikes were identified with Neuroexplorer. To the bursts containing exactly 2 spikes, we assigned a BD equal to their ISI and SN of 2. To single spikes, we assigned a BD of 2 ms and a SN of 1. The rationale for this procedure is as follows: (1) CNS neurons and particularly neocortical pyramidal neurons *in vivo* are tightly controlled by surrounding inhibition, and thus typically fire few spikes, and frequently single spikes (e.g., Pouille and Scanziani, 2001). A similar situation should be considered physiological in *in vitro* networks; (2) all units in which single spikes were occasionally observed were characterized by a majority of bursts containing two or more spikes, with an average SN always higher than 2; (3) the classical burst definition (SN ≥ 3) would lead to wrong estimates of SN; and (4) our networks were silent during the down-states. We discarded the units (1–2 in each network) that fired continuously. As is shown in the SN time histograms of Figures 2B,D only at the end of each burst the number of spikes becomes very small. On the contrary, the average SN values and their standard errors indicate that the cases of one or two spikes only are very rare.

**Figure 1:**
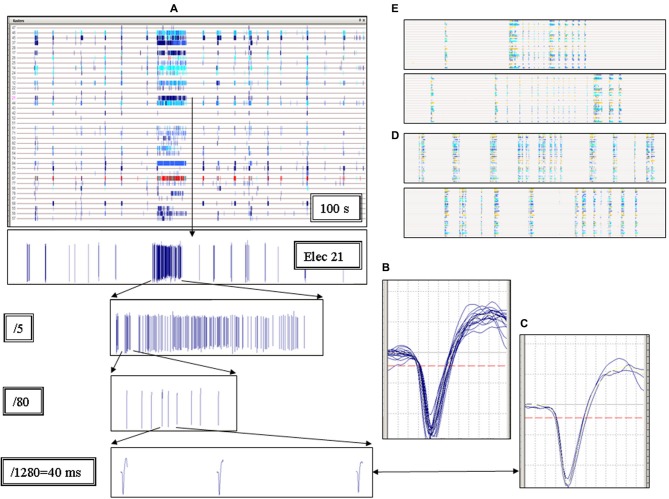
**Elementary properties of the short and long up-states. (A)** Each column represents an up-state (called network burst or global burst) and each row (here highlighted by a horizontal line) shows the trace recorded by one electrode. The panel shows a raster plot representing the multi-electrode activity recorded during 100 s. Each small vertical tick in a row is the timestamp representing a single spike. If the same electrode acquires spikes from more than one identified unit (neuron), the ticks corresponding to different units are indicated by different colors. Notice the very long burst in the middle of the raster plot. The spike waveforms recorded from electrode 21 are shown in the 1st, 2nd, 3rd and 4th bottom inset, at increasing time scale magnifications, as indicated. The last inset on the bottom shows 40 ms continuous recording. **(B–C)** Plots of the spike waveforms on a time-scale of 1.2 ms. The spikes shown in the 4th inset are superimposed in panel B and originated from the very long up-state. The spikes of all the very short network bursts following the long one are superimposed in panel C. Notice the similarity between all of these waveforms. **(D–E)** Raster plots representative of other experiments in Mutant cultures (duration and timescale were as in **A**).

**Figure 2:**
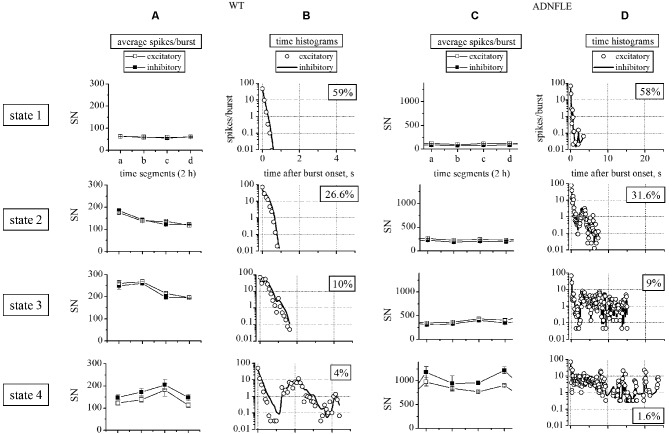
**Sorting of heterogeneous bursts into statistically different states**. The left **(A, B)** and right **(C, D)** panels correspond to WT and Mutant data, respectively. Rows show the properties of the four different states (see Section Materials and Methods), as indicated. **(A, C)** Plots of the different average number of spikes per burst (SN), calculated for each state within four 2 h segments. Open and closed squares indicate, respectively, excitatory and inhibitory neurons. **(B, D)** Plot of the different time histograms of the average spikes/burst (bin was 0.1 s) corresponding to the indicated states. Open circles and continuous lines indicate, respectively, excitatory and inhibitory neurons. In the WT network we identified 69 unit (52 excitatory and 17 inhibitory). The number of identified up-states in the four 2 h segments were, respectively, 736, 755, 758 and 980. The average IBIs (inter burst intervals) were comprised between 8 and 10 s. In the Mutant network we identified 78 unit (63 excitatory and 15 inhibitory). The number of identified up-states in the four 2 h segments were, respectively, 422, 256, 299 and 282. The average IBIs were comprised between 22 and 26 s.

The activity pattern of the global network was analyzed with standard (Ham et al., 2008) as well as more recent (Gullo et al., 2012) procedures. A running window of variable duration (5–100 ms) was used to locate the start of the up-state and collect the spikes. The low- and high-thresholds and the minimum interburst length were adjusted in each experiment, in order to correctly acquire both short and long bursts. The new procedure (Gullo et al., 2012) classified the network states by a PCA based on the SN time histogram, the neuron number and BD.

### Statistical tests

**Figure 3:**
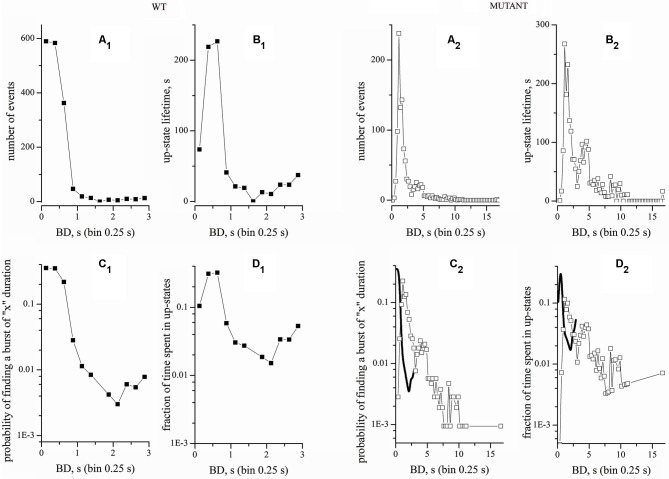
**Comparison of raw and normalized recordings from WT and Mutant cultures**. Left and right panels correspond to data obtained from WT and Mutant, as indicated. The four upper panels correspond to raw data. The four lower panel correspond to normalized data. Bin width was 0.25 s. **(A_1_, A_2_)** BD histograms. **(B_1_, B_2_)** Plots of up-states lifetimes. **(C_1_, C_2_)** Normalized BD histograms. **(D_1_, D_2_)** Fraction of time spent in up-states. In **(C_2_)** and **(D_2_)**, the thick lines (B-Splines) indicate the WT data. WT and Mutant experiments had durations of 9 and 4 h, respectively. The total number of bursts and the total up-state time (given within brackets) were 1661 (702 s) and 1058 (2320 s) for WT and Mutant experiments, respectively.

**Figure 4:**
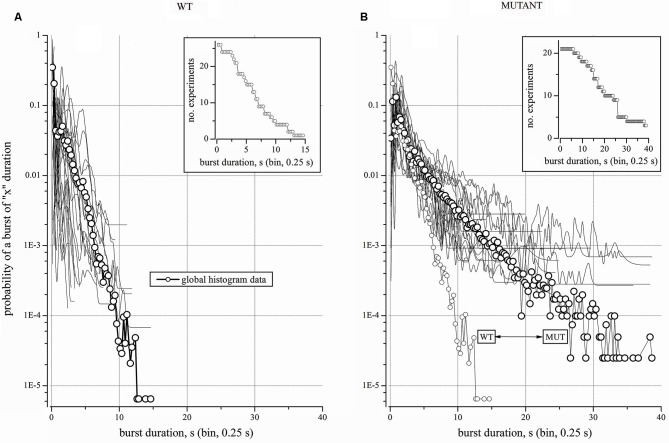
**Properties and statistics of WT and Mutant experiments**. **(A)** WT. Log-scale plot of the average probability of finding a burst of “x” duration in 27 experiments (line + open circles). To show the heterogeneity of networks, the 27 single-experiment plots are superimposed as thin lines (points are connected by B-Spline). Inset: distribution of the burst durations (BD) for the experiments that were considered in **(A)**. **(B)** Mutant. Log-scale plot of the average probability of finding a burst of “x” duration in 21 experiments (line + open circles). To illustrate the heterogeneity of networks, the 21 single-experiment plots are superimposed as thin lines (points are connected by B-Spline). Inset: distribution of the BD for the experiments that were considered in **(B)**.

**Figure 5:**
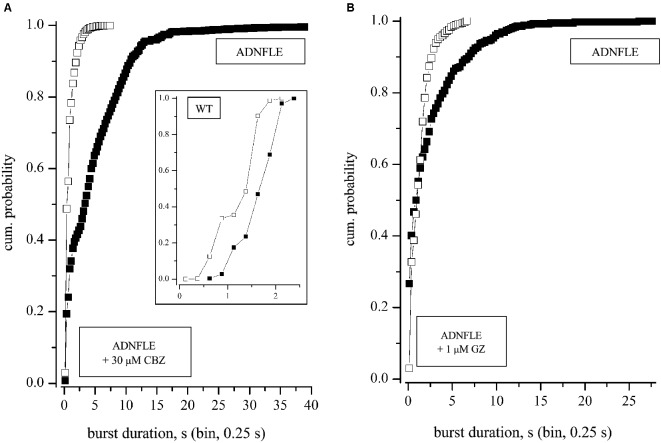
**Effects of CBZ and GZ. (A)** Superimposed cumulative histograms of the BD in the absence (control) and in the presence of 30 μM CBZ. Data refer to a typical experiment in which CBZ was applied for 8 h, after 8 h recording in control conditions. The inset shows the result of an exemplary experiment (1 out of 4) performed in WT networks (same symbols and scales as used for Mutant). **(B)** Superimposed cumulative histograms of the BD in the absence (2 h; control) and in the presence of 1 μM GZ (2 h). The networks recovered the initial level of activity, after washout (not shown). For both CBZ and GZ, data are representative of 3 experiments carried out on mice from different litters. In both A and B the application of the non-parametric Kruskal-Wallis tests resulted in *p*-values < 0.0001.

Data are given as mean values ± standard error of the mean, with *n* indicating the number of experiments. Statistical significance for normally distributed data was assessed with OriginPro 8.0 (OriginLab Co., Northampthon, MA), by using ANOVA test with the Bonferroni *post-hoc* *p*-values. Normality was tested by the Kolmogorov-Smirnov test. Non-normally distributed data were analyzed with non parametric tests, by using the XLSTAT-Pro software (Addinsoft, USA). In Figure 2 we applied the Mann-Whitney, the Wilcoxon Signed Rank and Kruskal-Wallis tests, which gave similar results. The BD histograms from different experiments (Figures 3–5) generally did not present an identical number of data points (bins). Therefore, to apply the Wilcoxon-Sign test, the bin number was uniformed by adding zeros in empty bins. Instead, to directly compare the real BDs over many time segments, we used the Kruskal-Wallis test (the non-parametric equivalent of ANOVA). The data heterogeneity (e.g., Figure 4) was further characterized by multiple comparison methods such as the Dunn’s and the Steel-Dwass-Critchlow-Finger’s (with Bonferroni correction; Hollander and Wolfe, 1999).

## Results

We prepared our neuronal cultures from transgenic mice expressing β2-V287L. These mice simulate a heterozygous condition and display spontaneous seizures, as discussed in Materials and Methods. The littermates not expressing the transgene were used as controls. It is important to note that expression of the transgene does not alter the overall expression of heteromeric nAChRs onto the plasma membrane (Manfredi et al., 2009). For briefness, we denote the neuronal cultures dissociated from mice expressing or not β2-V287L, as Mutant and WT, respectively.

### Qualitative characterization of the mutant network firing

Our MEAs allow the simultaneous recording of approximately 2% of the neurons implicated in the global network activity (Gullo et al., 2009). The firing activity of individual neurons is conveniently illustrated as “time raster plots”, i.e., temporal sequences of the spikes (displayed as ticks) recorded from each identified neuron. Different colors are used if more than one neuron is sampled by the same electrode. Figure 1A shows a typical 100 s raster plot from a Mutant culture. Each horizontal trace represents continuous recording from one active electrode. The apparent “columns” reveal the network up-states, i.e., the periods during which most neurons fire simultaneously. The thin columns are normal short up-states, whereas the wide column in the middle reveals an up-state lasting ~10 s. This latter was a seizure-like event. Long up-states such as this were consistently observed in our Mutant cultures, interspersed among the normal (i.e., similar to the WT’s) bursting activity. The bottom panels of Figure 1A show the spike waveform from a single electrode at progressively smaller time-scales. The three spikes shown in the last panel belong to the seizure-like event and are superimposed in Figure 1C, which shows their shapes were virtually identical. Conversely, in Figure 1B we superimposed all of the spikes that followed the long seizure-like event. On average, the waveforms in B and in C were not significantly different, which indicates that the neurons fire similar action potentials during the short and long up-states. The independency of the spike waveform of the up-state duration suggests that the latter depends on changes of synaptic dynamics and not on intrinsic neuron excitability. In Mutant cultures, the up-state duration was heterogeneous. Examples are shown in Figures 1D,E, and more will be discussed in the context of our pharmacological tests. These results suggest that heterogeneity of seizure-like events is a typical characteristic of networks expressing β2-V287L. This qualitative conclusion is quantified below.

### Heterogeneous up-states in WT and mutant networks

The up-states in cultured neocortical networks can be assigned to four statistically distinct groups, based on SN and BD (Gullo et al., 2012). We applied a similar procedure to our cultures from transgenic mice. Figure 2 plots the results of this analysis for the excitatory and inhibitory neurons in two representative WT and Mutant cultures, as indicated. For each experiment, SN and BD were computed in consecutive time segments of 2 h which contained on the order of 10^5^ spikes, which warrants stationarity. As is shown in Figures 2A (WT) and 2C (Mutant), the average SN per burst (i.e., the average SN of all the recorded units, during an up-state) increased from the 1st state to the 4th state, and the difference was particularly marked in the Mutant networks (notice the different y-scale in WT and Mutant panels). In general, the number of excitatory neurons engaged in up-states increased in the states characterized by longer BDs, during which neurons were more likely to be re-recruited. In particular, approximately 60% of the excitatory neurons were engaged in the 1st state, whereas almost all of them were engaged in the 4th state. On the other hand, all of the inhibitory neurons were always engaged in the up-states (not shown).

The spike distribution within bursts was studied by computing the time histograms for each state. These data are plotted in Figures 2B (WT) and 2D (Mutant), which show a clear heterogeneity of the four states. More importantly, an increase in the average BD was observed between the WT and Mutant experiments in all the four states (notice the different time scales of the corresponding panels). In particular, a dramatic difference was observed in the maximal BD, which changed from 5 s in the WT to 25 s in the Mutant. It is interesting to notice that the latter duration is very similar to the average seizure duration in living mice (S3 line; Manfredi et al., 2009).

### Up-state lifetimes in WT and mutant networks

A problem of data normalization arises when comparing WT and Mutant experiments with different durations. Detecting or not a rare seizure-like event depends on the recording time. Hence, we compared the results of normalizing our data by the number of up-states or by the total experiment time. An example is given in Figure 3, which plots the BD histograms for a WT (left panels) and a Mutant network (right panels, as indicated). These cultures were prepared from the same mice litter and recording was made at the same time in culture. First, we simply computed the histograms of the number of events (Figures 3A_1_,A_2_). Because the up-state duration was highly variable, we also plotted the total time spent by the network in up-states of duration corresponding to each bin (Figures 3B_1_,B_2_). Next, we normalized the number of events histograms to the total number of events in each record (Figures 3C_1_,C_2_). Finally, we normalized the up-state lifetimes by the total experimental time (Figures 3D_1_,D_2_). In other words, we computed the fraction of the total events within a certain duration bin and the fraction of time spent in different up-states. Once again, notice the different time scales for WT and Mutant networks. In the latter, we observed a significant fraction of up-states with long duration (>10 s), which were totally absent in WT cultures. For immediate comparison of WT and Mutant, Figures 3C_1_,D_1_ also report the WT data (thick lines) taken, respectively, from Figures 3C_1_,D_1_.

The burst distribution has a broadly exponential shape, with a large prevalence of short events. However, multiple peaks are present in the burst distribution of the Mutant network, consistently with the higher burst heterogeneity apparent in Figure 2. This result suggests that different patterns of connectivity strength are possible during activity. Analogous results were obtained in 12 WT and 68 Mutant experiments and tested with several non-parametric statistical methods, which gave *p*-values << 0.0001 between WT and Mutant cultures. In particular, no burst events were ever observed in Mutant networks with durations between 4 and 16 s.

### Burst events’ distribution

Because the above normalization procedures gave similar results, the rest of our experiments is illustrated by plotting the probability of a burst of a given duration (as in Figures 3C_1_,C_2_). We first analyzed 27 WT networks (Figure 4A), for a grand-total of more than 20 days of recording. Each experiment contained at least 90 active neurons. The mean segment duration was 66,543 ± 4035 s, with a mean number of bursts of 3484 ± 2042 and an average BD of 0.75 ± 0.19 s (with IBI ~19 s). In Figure 4A, continuous lines correspond to the 27 individual histograms, whereas the global histogram is shown as thick line with open circles. The fast “decay” of the distribution shows that long bursts were extremely rare. This is also indicated by the inset, which plots the number of experiments vs. BD (bin width of 0.25 s). During a total recording time of 504 h (comprising 94,669 events), we observed only one up-state lasting more than 15 s. Therefore, the probability of occurrence of the very long up-states in the WT networks was in the order of 10^−5^. The heterogeneity of these experiments was analyzed with a Kruskal-Wallis test applied to the 27 WT experiments plus the average histogram. These populations were statistically different (*p* < 0.0001). The Dunn/bilateral test analysis (with the Bonferroni correction of 0.0001) indicates that there were nine groups and the 351 multiple pair comparisons of the average distribution probability with the other 27 data sets resulted to have a *p* > 0.05 only once.

In contrast, the Mutant networks (21 independent experiments from 8 litters), revealed BDs of up to 40 s (Figure 4B) and the global distribution (large circles; the total recording time was 167 h) decayed much more slowly than the WT’s. The inset gives the number of experiments vs. BD. For easier comparison, Figure 4B also gives the global distribution of the WT experiments (small circles). The probability of observing very long up-states in Mutant cultures was two orders of magnitude higher than in the WT. The WT and Mutant distributions resulted to be significantly different when tested with both the Wilcoxon and the Mann-Whitney method (*p* << 0.0001). Once again, the Kruskal-Wallis analysis on the Mutant experiments plus the global average data indicates that these populations were statistically different (*p* < 0.0001). The Dunn/bilateral test analysis (with the Bonferroni correction of 0.0001) defines four distinct groups. The multiple pair comparisons of the average distribution with those of the other 21 experiments gave *p* > 0.05 for eight data groups, whereas the other 13 experiments had *p* < 0.05. Thus, the Mutant data turned out to be somewhat more uniform than the WTs.

The overlap of WT and Mutant data was tested by comparing the two global distributions with each individual experiment of the opposite type, i.e., the Mutant-global distribution was compared with each WT experiment, and *vice versa*. All the Mutant experiments were statistically different from the WT-global distribution (*p* was always << 0.0001; Kruskal-Wallis test). The reciprocal test shows that only one WT gave *p* > 0.05, while the others gave *p* << 0.0001.

### The effect of CBZ

CBZ inhibits the voltage-dependent Na^+^ currents (McLean and Macdonald, 1986; Schwarz and Grigat, 1989; Kuo et al., 1997) and heteromeric nAChRs (Picard et al., 1999; Hoda et al., 2009; Di Resta et al., 2010). It can also potentiate GABA_A_ receptors (Liu et al., 2006b). The effective concentrations *in vitro* are between 1 and 100 μM. The reported concentrations of CBZ in the cerebrospinal fluid of treated patients are in the range 5–50 μM (McLean and Macdonald, 1986; Oby et al., 2006). In WT networks, CBZ blocked firing activity with an IC_50_ of 49 ± 5.6 μM (*n* = 4). A typical experiment is shown in the inset to Figure 5A. The drug was even more effective in Mutant networks, in which 50% inhibition of the firing activity was observed with 10 μM CBZ. Figure 5A illustrates the results of applying 30 μM CBZ to Mutant networks recorded for 12/24 h. The drug strongly decreased the mean BD, as is shown by comparing the cumulative distribution of BDs before (filled squares) and during (open squares) CBZ application. Before CBZ application, this network showed 10 seizure-like events with BDs in the range from 9 to 10 s, and 7 events with BDs from 10 to 17 s. In the presence of CBZ, no seizure lasted longer than 8 s and only 18 and 7 seizures were observed, respectively, in the time segments from 6 to 7 s and from 7 to 8 s. No alteration of the spike waveform was produced by CBZ on Mutant networks (not shown).

### The effect of modulating GABAergic transmission

Because previous evidence in animal models of ADNFLE has pointed to alterations in the neocortical GABAergic transmission, we studied the effect of several GABA_A_R ligands on our cultures. As positive modulator we used the benzodiazepine midazolam (MDZ; from 0.3 to 100 nM). As negative modulators, we used: (1) gabazine, a selective antagonist of both phasic and tonic GABA_A_Rs, at concentrations above 0.5 μM (Stell and Mody, 2002; Farrant and Nusser, 2005); and (2) penicillin-G (500 μM), a selective antagonist of phasic GABA_A_Rs (Yeung et al., 2003). As expected, MDZ inhibited the bursting activity of both WT and Mutant networks, at concentrations lower than 30 nM. These results are analogous to those we have extensively characterized recently (e.g., see Figure 7 in Puia et al., 2012), and will not be reproduced here. Higher doses completely silenced the network (not shown). Somewhat surprisingly, however, the GABA_A_ antagonists also tended to decrease the Mutant network activity. The results of a typical experiment with GZ (1 μM) are shown in Figure 5B. The cumulative BD distributions show that GZ inhibited the production of long bursts. Altogether, all of the compounds we tested tended to decrease either BD or the frequency of the long up-states, or both. The effects were reversible on washout.

## Discussion

Our neocortical cultures constitute a model of spontaneous hyperexcitability *in vitro*. No sign of abnormal firing activity was detected in WT cultures, in which the occurrence of prolonged up-states was extremely rare. In contrast, the neuronal networks expressing β2-V287L, besides displaying considerably higher BDs, systematically generated prolonged synchronized bursts, with durations of 20–30 s. Moreover, no distortion of the action potential waveform was observed in the cultures expressing β2-V287L, which suggests that the transgene mainly affects the excitatory/inhibitory synaptic dynamics. Although a detailed quantification of the relative proportion of excitatory and inhibitory neurons was not carried out in the present work, our previous results indicate that in our cultures the excitatory/inhibitory balance is generally close to the one observed in the neocortex (Gullo et al., 2010; Becchetti et al., 2012). Overall, the interpretation of our results we favour is that β2-V287L alters the local connectivity during synaptogenesis. This interpretation is consistent with previous observations *in vivo*. In fact, although β2-V287L needs to be expressed throughout brain development for the epileptic phenotype to arise, no overt morphological alteration is observed in the brain of transgenic mice (Manfredi et al., 2009). From an epileptologic standpoint, a direct comparison cannot be drawn between the long up-states observed *in vitro* and the seizures recorded in transgenic mice, especially because the cerebral cortex undergoes very complex regulation by extra-cortical afferents, such as the thalamic. Nonetheless, the spontaneous nature of the long bursts observed *in vitro*, their low probability and the similarity between their duration and the average duration of seizures in transgenic mice (Manfredi et al., 2009) suggest that the abnormal excitability of the neocortical cultures reproduces some features of the *in vivo* seizures.

Determining the ADNFLE mechanisms is not straightforward because, in the frontal cortex, heteromeric nAChRs regulate both glutamate and GABA release (Gioanni et al., 1999; Alkondon et al., 2000; Lambe et al., 2003; Couey et al., 2007; Dickinson et al., 2008; Aracri et al., 2010, 2013; Marchi and Grilli, 2010). The well-known sensitivity of neuronal excitability to the GABAergic tone has led to hypotesize that an altered nAChR-dependent modulation of the GABAergic system may facilitate seizures. One possibility is that higher GABA release tends to synchronize pyramidal neurons, by inducing a post-inhibitory activity “rebound” caused by de-inactivation of low-threshold calcium channels and pacemaker HCN-type channels (Klaassen et al., 2006). In addition, increased reciprocal inhibition between interneurons (particularly basket cells in layer V) could lead to network disinhibition (Aracri et al., 2010). Regardless of the specific effect(s), nAChRs could regulate GABAergic transmission in at least two ways (not mutually exclusive). First, nAChRs are known to regulate the development of the excitatory/inhibitory balance by regulating the maturation of both GABAergic transmission (Liu et al., 2006a) and dendritic spines (Lozada et al., 2012), during brain development. Second, because ADNFLE-linked mutations often potentiate the nAChR function, they may cause direct nAChR-dependent hyperexcitability of the mature neocortex. Our results, although by no means excluding the second mechanism, suggest that the first mechanism is operating in our mice. In fact, hyperexcitability persists after the neocortex is dissociated and then reconstituted *in vitro*. Under these conditions, the cholinergic fibers which regulate the nAChRs *in vivo* are eliminated. Another source of ACh in the neocortex is constituted by populations of intrinsic cholinergic cells (e.g., Semba, 2004; von Engelhardt et al., 2007). Hammond and colleagues recently reported that a significant cholinergic tone is in fact maintained in rat neocortical cultures grown on MEA dishes (Hammond et al., 2013). Intrinsic cholinergic neurons are also present in the murine neocortex. However, in the cortical region where they reach the highest density (the somatosensory), these cells appear between P4 and P6 and reach a peak during the second postnatal week (Consonni et al., 2009). In agreement with this notion, applying DHβE (1 μM) to inhibit the heteromeric nAChRs produced negligible effects on the excitability of our WT and Mutant networks (data not shown). Therefore, the spontaneous nAChR activation is presumably low, in our cultures.

In transgenic mice, CBZ is poorly effective in controlling the epileptic phenotype (Manfredi et al., 2009). In contrast, the drug turned out to be much more effective *in vitro*. CBZ is known to inhibit voltage-gated Na^+^ channels, by slowing-down the recovery from inactivation, with an IC_50_ of approximately 50 μM (McLean and Macdonald, 1986; Schwarz and Grigat, 1989; Kuo et al., 1997). However, at the concentrations we tested in cultures expressing the transgene, no effect was produced on the action potential shape. More recent evidence indicates that CBZ blocks heteromeric nAChRs and potentiates GABA_A_ receptors, at concentrations larger than 5 μM (nAChRs; Picard et al., 1999; Di Resta and Becchetti, 2013). Therefore, we interpret our results as suggesting that in neocortical cultures CBZ exert its effects on synaptic transmission at doses at which the effect on voltage-gated Na^+^ channels is weak. Hence, targeting either heteromeric nAChRs or GABA_A_ receptors, or both, may be an effective therapeutic strategy in ADNFLE. Our pharmacological results are also consistent with the observation that, in some murine models of ADNFLE, expression of the transgene leads to higher GABA release (e.g., Klaassen et al., 2006), which may lead to hyperexcitability by the mechanisms discussed earlier. If this is the case, a partial inhibition of GABA_A_Rs should dampen the effect, which is what we observed with low doses of GABAergic antagonists. On the other hand, potentiating GABA release beyond the effect due to β2-V287L with benzodiazepines is expected to first remove the epileptiform firing events (at lower concentrations) and then completely silence the network (at higher concentrations).

At the present stage, we cannot better define the mechanism of hyperexcitability. Nonetheless, our experimental model complements the brain slice preparation in that it allows (i) to determine the role of β2-V287L on synaptic formation; and (ii) to study the effect of sustained applications of pharmacological modulators on the balance of excitatory and inhibitory transmission. Besides the specific relevance for ADNFLE, experimental approaches such as the one illustrated here will be invaluable to carry out preliminary screens of new drugs as well as studying the sustained effects of AEDs in neuronal networks.

## Conflict of interest statement

The authors declare that the research was conducted in the absence of any commercial or financial relationships that could be construed as a potential conflict of interest.
